# Ukrainian Plant Trait Database: UkrTrait v. 1.0

**DOI:** 10.3897/BDJ.12.e118128

**Published:** 2024-02-13

**Authors:** Denys Vynokurov, Dariia Borovyk, Olha Chusova, Anastasia Davydova, Denys Davydov, Jiří Danihelka, Iwona Dembicz, Svitlana Iemelianova, Ganna Kolomiiets, Ivan Moysiyenko, Viktor Shapoval, Oleksandr Shynder, Nadiia Skobel, Oksana Buzhdygan, Anna Kuzemko

**Affiliations:** 1 Department of Geobotany and Ecology, M.G. Kholodny Institute of Botany, National Academy of Sciences of Ukraine, Kyiv, Ukraine Department of Geobotany and Ecology, M.G. Kholodny Institute of Botany, National Academy of Sciences of Ukraine Kyiv Ukraine; 2 Department of Plant Biology and Ecology, University of the Basque Country (UPV/EHU), Leioa, Spain Department of Plant Biology and Ecology, University of the Basque Country (UPV/EHU) Leioa Spain; 3 Department of Botany and Zoology, Faculty of Science, Masaryk University, Brno, Czech Republic Department of Botany and Zoology, Faculty of Science, Masaryk University Brno Czech Republic; 4 Department of Taxonomy, Institute of Botany of the Czech Academy of Sciences, Průhonice, Czech Republic Department of Taxonomy, Institute of Botany of the Czech Academy of Sciences Průhonice Czech Republic; 5 Institute of Environmental Biology, Faculty of Biology, University of Warsaw, Warsaw, Poland Institute of Environmental Biology, Faculty of Biology, University of Warsaw Warsaw Poland; 6 Department of Population Ecology, Institute of Botany of the Czech Academy of Sciences, Průhonice, Czech Republic Department of Population Ecology, Institute of Botany of the Czech Academy of Sciences Průhonice Czech Republic; 7 Department of Botany, Kherson State University, Kherson, Ukraine Department of Botany, Kherson State University Kherson Ukraine; 8 Falz-Fein Biosphere Reserve "Askania-Nova" NAAS of Ukraine, Askania-Nova, Ukraine Falz-Fein Biosphere Reserve "Askania-Nova" NAAS of Ukraine Askania-Nova Ukraine; 9 M.M. Gryshko National Botanical Garden, National Akademy of Sciences of Ukraine, Kyiv, Ukraine M.M. Gryshko National Botanical Garden, National Akademy of Sciences of Ukraine Kyiv Ukraine; 10 Theoretical Ecology, Institute of Biology, Freie Universität Berlin, Berlin, Germany Theoretical Ecology, Institute of Biology, Freie Universität Berlin Berlin Germany

**Keywords:** flora, life form, life span, phenology, plant characteristics, seed mass, specific leaf area, Ukraine, vascular plants

## Abstract

**Background:**

Considering the growing demand for plant trait data and taking into account the lack of trait data from Eastern Europe, especially from its steppic region, we launched a new Ukrainian Plant Trait Database (UkrTrait v. 1.0) aiming at collecting all the available plant trait data from Ukraine.

To facilitate further use of this database, we linked the trait terminology to the TRY Plant Trait Database, Thesaurus of Plant Characteristics (TOP) and Plant Trait Ontology (TO). For taxa names, we provide the crosswalks between the Ukrainian checklist and international sources, i.e. GBIF Backbone Taxonomy, World Checklist of Vascular Plants (World Checklist of Vascular Plants (World Checklist of Vascular Plants (WCVP), World Flora Online (WFO) and Euro+Med PlantBase. We aim to integrate our data into the relevant global (TRY Plant Trait Database) and pan-European (FloraVeg.EU) databases. The current version of the database is freely available at the Zenodo repository and will be updated in the future.

**New information:**

Until now, plant traits for the Ukrainian flora were scattered across literature, often focusing on single species and written mainly in Ukrainian. Additionally, many traits were in grey literature or remained non-digitised, which rendered them inaccessible to the global scientific community. Addressing this gap, our Ukrainian Plant Trait Database (UkrTrait v. 1.0) represents a significant step forward. We compiled and digitised plant traits from local Ukrainian literature sources. Furthermore, we performed our own field and laboratory measurements of various plant traits that were not previously available in literature. In the current version of the UkrTrait, we focus on vascular plant species that are absent from the other European trait databases, with emphasis on species that are representative for the steppe vegetation. Traits assembled from literature include life span (annuals, biennials, perennials), plant height, flowering period (flowering months), life form (by Raunkiaer), plant growth form and others. Our own measured traits include seed mass, seed shape, leaf area, leaf nitrogen concentration and leaf phosphorus concentration. The current version, i.e. UkrTrait v. 1.0, comprises digitised literature data of 287,948 records of 75 traits for 6,198 taxa and our own trait measurements of 2,390 records of 12 traits for 388 taxa.

## Introduction

Plant traits, measured at the species level, reflect plants' performance in response to abiotic and biotic environmental constraints (Violle et al. 2007). Using species-specific traits together with the community composition data allows one to upscale the assessment of functioning from the species level to the entire community scale and allows one to directly look at the whole-community relationships between entire species assemblages and the respective functional processes carried out in these communities in response to environmental drivers (Grime 1977, Westoby 1998, Wright et al. 2004, Díaz et al. 2016, Pierce et al. 2017, Weigelt et al. 2021, Carmona et al. 2021). Trait-based approaches, acknowledged by ecologists as the common framework for the comparison of species or community functioning amongst any combination of communities within and between ecosystems and habitat types (Laureto et al. 2015), are widely adopted for testing biodiversity-ecosystem functioning relationships (Grossman et al. 2018). They are also used to link plant functioning to the diversity and performance of higher trophic levels (Schuldt et al. 2019) as well as to the whole-ecosystem functioning (De Bello et al. 2010) and ecosystem services (Diaz et al. 2007). Additionally, these approaches are used to predict the functional consequences of biodiversity change (Lavorel and Garnier 2002) and to provide information for ecosystem management and conservation policies on the altered biodiversity (Kissling et al. 2018, Perrino et al. 2022).

In response to the high potential of plant traits for their application to both theoretical and applied research, a number of regional and global plant trait databases were established in recent decades, such as BIOPOP (Poschlod et al. 2003), BROT (Tavşanoğlu and Pausas 2018), TRY (Kattge et al. 2020), AusTraits (Falster et al. 2021), PADAPT (Sonkoly et al. 2023), GRooT (Guerrero‐Ramírez et al. 2021) and RSIP (Tumber‐Dávila et al. 2022). While these trait databases fill in numerous gaps in global coverage and provide an important basis for both regional studies and global synthesis projects, there are still strong limitations in trait availability for plant species that are endemic to Eastern Europe . Ukrainian flora, particularly that of the steppe zone, is strongly under-represented in the plant trait literature.

According to the latest available nomenclature checklist (Mosyakin and Fedoronchuk 1999), Ukrainian flora included about 6,000 taxa of native and frequently cultivated vascular plants, including hybrids and infraspecific taxa. The most species-rich parts of Ukraine are the Carpathian Mountains and Crimea, harbouring ca. 2,000 and 2,250 species, respectively (Dobrochaeva 1987, Mosyakin and Fedoronchuk 1999). Around 850 species of the Ukrainian flora are specifically associated with steppe vegetation, 400 species with limestone and chalk outcrops, 300 with the Crimean mountain forests, 150 with alluvial sand deposits and 100 with outcrops of granite rocks (Dobrochaeva 1987). These vegetation types contain a particularly large number of endemic species and of the species not occurring in Central and Western Europe. Consequently, many of these species are missing from the existing plant trait databases. Considering the growing demand for plant trait data and, at the same time, the lack of trait data from Eastern Europe, we launched the Ukrainian Plant Trait Database (UkrTrait v.1.0), aiming at filling in the gaps in the traits' availability for plant species of the Ukrainian flora.

## Project description

### Title

Establishing the Ukrainian Plant Trait Database

### Personnel

Project PI: Denys Vynokurov; project members: Dariia Borovyk, Olha Chusova, Anastasia Davydova and Denys Davydov. Experts involved in the project: Oksana Buzhdygan, Jiří Danihelka, Svitlana Iemelianova, Ganna Kolomiiets, Anna Kuzemko, Ivan Moysiyenko, Viktor Shapoval, Oleksandr Shynder and Nadiia Skobel.

### Study area description

Ukraine

### Funding

The Ukrainian Plant Trait Database was established as part of the project 'Functional, syntaxonomical and phylogenetic diversity of steppes of Ukraine as a basis of an evaluation of their ecosystem services', supported by Grant of the National Academy of Sciences of Ukraine to research laboratories/groups of young scientists of the National Academy of Sciences of Ukraine for conducting research in priority areas of science and technology. The publication of this work has been supported by the Biodiversity Community Integrated Knowledge Library (BiCIKL) project, which receives funding from the European Union's Horizon 2020 Research and Innovation Action under grant agreement No 101007492.

## Sampling methods

### Study extent

The Ukrainian Plant Trait Database (UkrTrait v. 1.0) includes both traits assembled from literature sources and those measured in field and laboratory conditions. The nomenclature of the database was harmonised, based on the Ukrainian Checklist (Mosyakin and Fedoronchuk 1999) with crosswalks to the international taxonomical sources. Laboratory and field-trait measurements were done according to the standardised protocols for trait collection.

### Sampling description

First, we compiled the available plant traits using various published literature sources (Bordzilovsky 1938, Bordzilovsky and Lavrenko 1940, Kotov and Barbarych 1950, Kotov 1952, Klokov and Visiulina 1953, Zerov 1954, Klokov and Visiulina 1955, Kotov and Barbarych 1957, Kotov 1960, Kotov 1961, Visiulina 1962, Visiulina 1965, Dobrochaeva 1987, Protopopova 1991, Golubev 1996, Didukh 2000, Didukh 2002, Didukh 2004, Didukh 2007, Didukh 2010), unpublished sources (i.e. manuscripts, theses, reports) and expert knowledge (Suppl. material 1). We digitised plant height for 4,889 taxa of Ukrainian flora; life span for 5,951 taxa; flowering period for 4,939 taxa; and other categorical traits (more details are given in the Step description).

We extracted information about the residence time status for alien species from Protopopova (1991) and Protopopova and Shevera (2015), i.e. time of introduction (residence time status), geographic origin and degree of naturalisation (Suppl. material 1). Time of introduction included two trait modalities: archaeophytes are alien species deliberately or accidentally introduced before the year 1500, while neophytes are alien species introduced deliberately or accidentally after the year 1500. For some neophyte species, we changed the status to "neophyte (doubtfully)" when, in several other sources (Golubev 1996, Brown et al. 2023, Euro+Med 2023), the species was reported as native. By the degree of naturalisation, five groups were distinguished by Protopopova and Shevera (2015), following Kornaś (1990). Cultivated and escaped from cultivation species are listed in the database according to the Ukrainian Checklist (Mosyakin and Fedoronchuk 1999). For native species, we added information on the protection status in Ukraine, including whether the species is listed in the Red Data Book of Ukraine (Ministry of Ecology and Natural Resources of Ukraine 2021) and its corresponding protection category (Suppl. material 1).

For plant traits that were not available in literature, we performed our own measurements, including seed mass (327 measurements, 282 species), seed shape (335 measurements, 286 species), leaf area (130 measurements, 112 species), leaf nitrogen concentration and leaf phosphorus concentration (196 measurements, 188 species) and specific leaf area (72 measurements, 68 species) (Suppl. material 2). All the measurements were done according to the standardised protocols for trait collection (Pérez-Harguindeguy et al. 2013). We primarily targeted plant species absent from other European databases of plant traits, with the main focus on species that are representative of steppe vegetation. Most of the samples for trait measurements were georeferenced.

The list of taxa names was initially harmonised according to the Ukrainian Checklist "Vascular Plants of Ukraine: A Nomenclatural Checklist" (Mosyakin and Fedoronchuk 1999), which contained 6,074 vascular plant taxa, including infraspecific taxa and hybrids. Then we supplemented the species list with an additional 110 taxa names, newly listed for Ukraine since the Checklist had been published (e.g. Peregrym and Kuzemko 2010, Nachychko et al. 2018, Novák and Zukal 2018, Shevchyk et al. 2018, Ljubka 2019, Mosyakin and Mandák 2020, Moysiyenko et al. 2022 etc.). All taxa names and their authorship were checked for possible misprints to avoid misinterpretations in further use. The original corresponding taxa names from the Ukrainian Checklist were preserved for each taxon in the UkrTrait database.

Finally, we matched the species list of the UkrTrait database to the international taxonomic and nomenclatural sources and trait terminology to the ontologies and terms in the TRY Plant Trait Database (detailed information in Step description).

### Quality control

We used R packages ‘tidyverse’ (Wickham et al. 2019) and OpenRefine v. 3.6.0 for the data quality control.

### Step description

1. Preparation of the dataset of plant traits from literature sources (Suppl. material 1):

1.1. Extraction of numerical and categorical traits from the Identification key of vascular plants of Ukraine (Dobrochaeva 1987): plant height, life span and flowering period for all species of Ukrainian flora.

1.2. Digitisation of the monograph "Biological flora of Crimea" (Golubev 1996): 36 categorical traits for all species of Crimean flora.

1.3. Gathering the information about Raunkiaer life form and plant growth form from published (Bordzilovsky 1938, Bordzilovsky and Lavrenko 1940, Kotov and Barbarych 1950, Kotov 1952, Klokov and Visiulina 1953, Zerov 1954, Klokov and Visiulina 1955, Kotov and Barbarych 1957, Kotov 1960, Kotov 1961, Visiulina 1962, Visiulina 1965, Dobrochaeva 1987, Protopopova 1991, Golubev 1996, Didukh 2000, Didukh 2002, Didukh 2004, Didukh 2007, Didukh 2010) and unpublished sources (own manuscripts, theses, reports). Standardisation of categories of life form and plant growth form and adding the missing values by expert knowledge.

1.4. Digitisation of the residence time status for alien species (archaeophyte or neophyte) from Protopopova (1991) and Protopopova and Shevera (2015). Checking the taxon origin in Ukraine (native vs. alien) using other sources (Golubev 1996, Brown et al. 2023, Euro+Med 2023). Correction of the 'neophyte' category into 'neophyte (doubtfully)' and 'archaeophyte' category into 'archaeophyte (doubtfully)' when, in several other sources, the species was reported as native.

1.5. Extraction of the cultivated species and escaped from cultivation species which are listed in the "Vascular Plants of Ukraine: A Nomenclatural Checklist" (Mosyakin and Fedoronchuk 1999).

1.6. Extraction of the protection categories of species from the Red Data Book of Ukraine (Ministry of Ecology and Natural Resources of Ukraine 2021).

2. Preparation of the Dataset of measured plant traits (Suppl. material 2) using the standardised protocols for trait collection (Pérez-Harguindeguy et al. 2013):

- Field measurements of generative and vegetative plant height.
- Collection, scanning and drying of plant leaves and collection of plant seeds.
- Laboratory measurements of dry leaf mass, nitrogen concentration (quantity of nitrogen in the leaf per respective unit dry mass), phosphorus concentration (ratio of the quantity of phosphorus in the leaf per respective unit dry mass), seed dimensions (length, width and thickness of a seed) and dry seed mass.
- Calculation of leaf area and specific leaf area using scanned leaf images, calculation of seed shape using seed dimensions.

3. Taxonomical harmonisation and linking plant names to other sources (Suppl. material 3):

3.1. Harmonisation of species data according to "Vascular Plants of Ukraine: A Nomenclatural Checklist" (Mosyakin and Fedoronchuk 1999). Checking taxa names and their authorship against the World Checklist of Vascular Plants (World Checklist of Vascular Plants (World Checklist of Vascular Plants (WCVP), Euro+Med PlantBase and International Plant Names Index (IPNI).

3.2. Matching the UkrTrait species list to the international checklists:

- Euro+Med PlantBase (Euro+Med 2023);
- GBIF Backbone Taxonomy (GBIF Secretariat 2023) using the GBIF Species Lookup Tool and manual correction for fuzzy matches;
- World Checklist of Vascular Plants (World Checklist of Vascular Plants (World Checklist of Vascular Plants (WCVP) using R package ‘rWCVP’ (Brown et al. 2023, Brown and Walker 2023) and expert check for fuzzy matches;
- World Flora Online (WFO) using R package ‘WorldFlora’ (Kindt 2023) and expert check for fuzzy matches.

4. Linking trait terminology of the UkrTrait database to the Thesaurus of Plant Characteristics (TOP), the Plant Trait Ontology (TO) and TRY Plant Trait Database.

## Geographic coverage

### Description

Ukraine

### Coordinates

44°23'11"N and 52°22'46"N Latitude; 22°08'13"E and 40°13'40"E Longitude.

## Traits coverage

**Figure 1.**
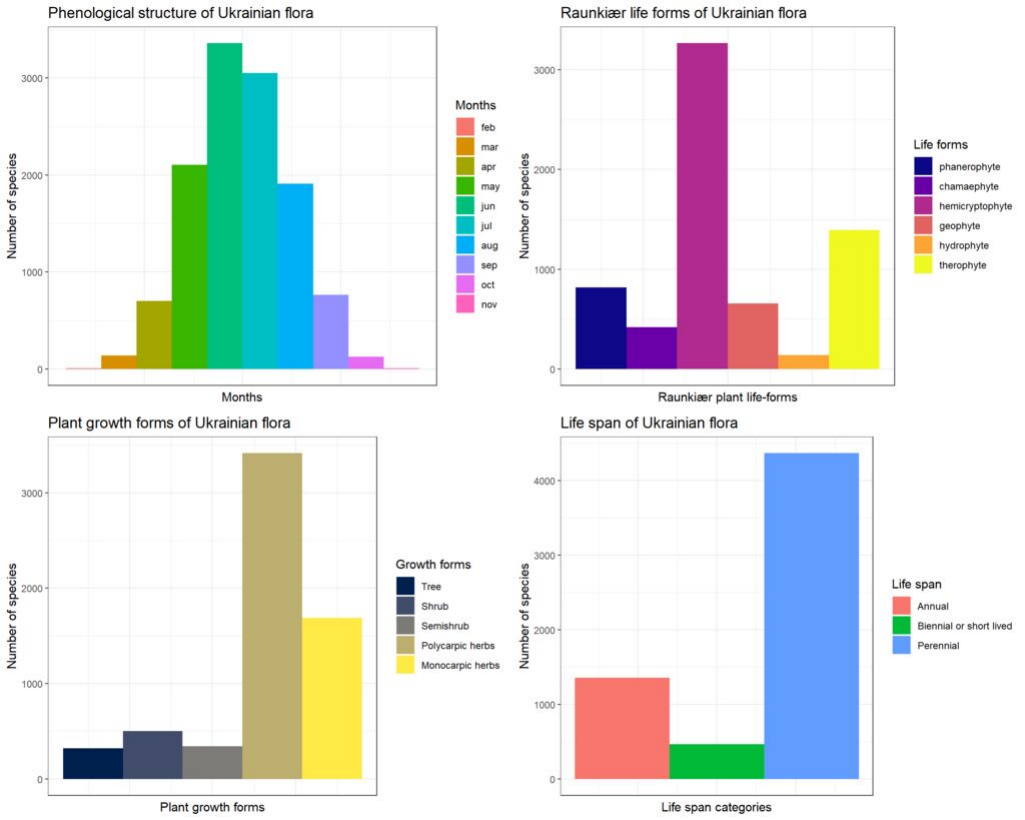
Bar charts for the selected categorical traits of Ukrainian flora: phenological structure, based on flowering period, Raunkiaer life forms, plant growth forms and life span.

**Figure 2.**
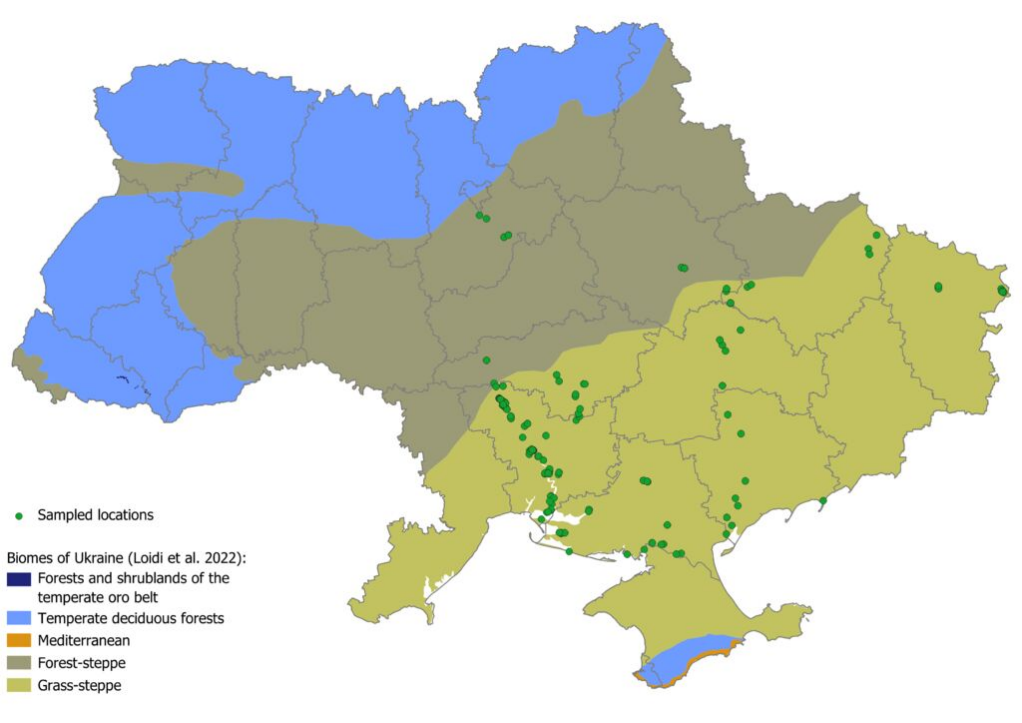
Map of Ukraine with borders of terrestrial biomes (Loidi et al. 2022) and locations where plant samples were collected for the trait measurements.

Overall, the Ukrainian Plant Trait Database (UkrTrait v.1.0) includes the literature-assembled data of 287,948 records for 75 traits for 6,198 plant taxa, as well as our own field and lab measurements of 12 traits for 388 species with in total of 2,390 records. The best-covered traits of Ukrainian flora are plant height, flowering period and life span, extracted from literature and also Raunkiaer life form and plant growth form, gathered based on both literature and expert knowledge (Fig. 1). All new trait measurements (including seed mass, seed shape, leaf area, leaf nitrogen concentration, leaf phosphorus concentration and others) were made on plants collected from the steppe biome, i.e. the forest-steppe and grass-steppe sub-biomes according to the Loidi et al. (2022) classification, within the following administrative regions in Ukraine: Dnipro, Kharkiv, Kherson, Kirovograd, Kyiv, Luhansk, Mykolaiv, Poltava and Zaporizhzhia (Fig. 2).

## Usage licence

### Usage licence

Open Data Commons Attribution License

## Data resources

### Data package title

Ukrainian Plant Trait Database UkrTrait v.1.0

### Resource link

https://zenodo.org/doi/10.5281/zenodo.10607076

### Number of data sets

3

### Data set 1.

#### Data set name

Dataset of measured plant traits

#### Description

Dataset is available in Suppl. material 2. The up-to-date version of the dataset can be found in the Zenodo repository.

**Data set 1.** 

Column label	Column description
UI	Unique Identifier.
ID_UkrTrait	ID of the species in the UkrTrait database.
taxon_UkrTrait	Taxon name in the UkrTrait database.
traitParameter	Measured parameter: height_gen_cm – generative plant height measured in the field (cm); height_veg_cm – vegetative plant height measured in the field (cm); leaf_area_mm2 – average one-sided projected surface area of the fresh leaf (mm^2^); leaf_mass_mg – average dry leaf mass (mg); sla_mm2_mg – average specific leaf area (SLA) – average one-sided area of the fresh leaf divided by its oven-dry mass (mm^2^ * mg^-1^); nitrogen_concentration – leaf nitrogen concentration, ratio of the quantity of nitrogen in the leaf per respective unit dry mass (mg * g^-1^); phosphorus_concentration – leaf phosphorus concentration, ratio of the quantity of phosphorus in the leaf per respective unit dry mass (mg * g^-1^); seed_dimension1_mm – length of a seed (mm); seed_dimension2_mm – width of a seed (mm); seed_dimension3_mm – thickness of a seed (mm); seed_shape – variance of its three dimensions, i.e. the length, the width and the thickness (unitless, raging from 0 to 1); seed_mass_mg – average dry mass of a seed (mg).
sampleNr	Sequence number of a measurement of the same parameter of the same plant species.
traitValue	Measured value.
locality	Place of collection of plant sample.
lat	Latitude (decimal degrees, WGS84).
lon	Longitude (decimal degrees, WGS84).
precision	Precision of coordinates (m).
date	Date in a format yyyy-mm-dd.
collector	Name(s) of collector(s).

### Data set 2.

#### Data set name

Dataset of plant traits from literature sources.

#### Description

Dataset is available in Suppl. material 1. The up-to-date version of the dataset can be found in the Zenodo repository.

**Data set 2.** 

Column label	Column description
ID_UkrTrait	ID of the species in the UkrTrait database.
taxon_UkrTrait	Taxon name in the UkrTrait database.
plantHeight_dobr	Average height of the whole plant (source: Dobrochaeva (1987)). Unit type: numerical (in cm).
flowerJan_dobr	Plant flowering period: flowering in January (source: Dobrochaeva (1987)). Unit type: binary (1 and 0).
flowerFeb_dobr	Plant flowering period: flowering in February (source: Dobrochaeva (1987)). Unit type: binary (1 and 0).
flowerMar_dobr	Plant flowering period: flowering in March (source: Dobrochaeva (1987)). Unit type: binary (1 and 0).
flowerApr_dobr	Plant flowering period: flowering in April (source: Dobrochaeva (1987)). Unit type: binary (1 and 0).
flowerMay_dobr	Plant flowering period: flowering in May (source: Dobrochaeva (1987)). Unit type: binary (1 and 0).
flowerJun_dobr	Plant flowering period: flowering in June (source: Dobrochaeva (1987)). Unit type: binary (1 and 0).
flowerJul_dobr	Plant flowering period: flowering in July (source: Dobrochaeva (1987)). Unit type: binary (1 and 0).
flowerAug_dobr	Plant flowering period: flowering in August (source: Dobrochaeva (1987)). Unit type: binary (1 and 0).
flowerSep_dobr	Plant flowering period: flowering in September (source: Dobrochaeva (1987)). Unit type: binary (1 and 0).
flowerOct_dobr	Plant flowering period: flowering in October (source: Dobrochaeva (1987)). Unit type: binary (1 and 0).
flowerNov_dobr	Plant flowering period: flowering in November (source: Dobrochaeva (1987)). Unit type: binary (1 and 0).
flowerDec_dobr	Plant flowering period: flowering in December (source: Dobrochaeva (1987)). Unit type: binary (1 and 0).
annual_dobr	Plant life span: belonging to annuals (source: Dobrochaeva (1987)). Unit type: binary (1 and 0).
biennialOrShortLived_dobr	Plant life span: belonging to biennials or short-lived plants (source: Dobrochaeva (1987)). Unit type: binary (1 and 0).
perennial_dobr	Plant life span: belonging to perennials (source: Dobrochaeva (1987)). Unit type: binary (1 and 0).
raunkiaer	Raunkiaer life form. Unit type: categorical (pha, cha, hem, geo, hydr, the). Explanation: Pha = phanerophyte, cha = chamaephyte, hem = hemicryptophyte, geo = geophyte, hydr = hydrophyte, the = therophyte. Should the species grow in several life forms, the main is shown in the first place and the rest are put into parentheses.
phanerophyte	Raunkiaer life form: belonging to phanerophytes. Unit type: binary (1 and 0).
chamaephyte	Raunkiaer life form: belonging to chamaephytes. Unit type: binary (1 and 0).
hemicryptophyte	Raunkiaer life form: belonging to hemicryptophytes. Unit type: binary (1 and 0).
geophyte	Raunkiaer life form: belonging to geophytes. Unit type: binary (1 and 0).
hydrophyte	Raunkiaer life form: belonging to hydrophytes. Unit type: binary (1 and 0).
therophyte	Raunkiaer life form: belonging to therophytes. Unit type: binary (1 and 0).
growth_form	Plant growth form. Unit type: categorical (tree, shrub, semishrub, herb_poli, herb_mono, liana_herb, liana_woody, epi). Explanation: tree = trees, shrub = shubs, semishrub = semishrubs, herb = herbs, herb_poli = polycarpic herbs, herb_mono = monocarpic herbs, liana_herb = herbaceous liana, liana_woody = woody liana, epi = epiphytic plants. Should the species grow in several growth forms, the main one is shown in the first place and the other ones are idicated in parentheses.
tree	Plant growth form: belonging to trees. Unit type: binary (1 and 0).
shrub	Plant growth form: belonging to shrubs. Unit type: binary (1 and 0).
semishrub	Plant growth form: belonging to semishrubs. Unit type: binary (1 and 0).
herb	Plant growth form: belonging to herbs. Unit type: binary (1 and 0).
herbPoli	Plant growth form: belonging to polycarpic herbs. Unit type: binary (1 and 0).
herbMono	Plant growth form: belonging to monocarpic herbs. Unit type: binary (1 and 0).
epiphyte	Plant growth form: belonging to epiphytes. Unit type: binary (1 and 0).
lianaWoody	Plant growth form: belonging to woody lianas. Unit type: binary (1 and 0).
lianaHerb	Plant growth form: belonging to herbaceous lianas. Unit type: binary (1 and 0).
speciesRange_golubev	Species geographic range (source: Golubev 1996). Unit type: categorical (cosm, crim, crim-adv, crim-anat, crim-balk, crim-balk-anat, crim-cau, crim-cau-anat, crim-cau-balk, crim-doubt, eeur-emed, emed, emed-was, eur, eur-emed, eur-med, eur-med-was, eur-wsib, euras-step, holar, kaz, med, med-euras-step, med-was, med-was-euras-step, NA, pal, pont, pont-end, pont-kaz, spal, was, was-euras-step, wmed, wpal). Explanation: med - Mediterranean; emed - Eastern Mediterranean; crim-cau-anat - Crimean-Caucasian-Anatolian; crim-balk-anat - Crimean-Balkan-Anatolian; crim-cau-balk - Crimean-Caucasian-Balkan; crim-balk - Crimean-Balkan; crim-anat - Crimean-Anatolian; crim-cau - Crimean-Caucasian; crim - Crimean endemic; crim-doubt - Crimean endemic doubtful; was - Western Asian; med-was - Mediterranean-Western Asian; emed-was - Eastern Mediterranean-Western Asian; eur-med - European-Mediterranean; eur-emed - European-Eastern Mediterranean; eur-med-was - European-Mediterranean-Western Asian; eeur-emed - Eastern Euroepan-Eastern Mediterranean; euras-step - Eurasian Steppic; eur-wsib - European-Western Siberian; pont - Pontic; pont-end - Pontic endemic; kaz - Kazakhstan; pont-kaz - Pontic-Kazakhstan; med-euras-step - Mediterranean-Eurasian Steppic; was-euras-step - Western Asian and Eurasian Steppic; med-was-euras-step - Mediterranean-Western Asian and Eurasian steppic; holar - Holarctic; pal - Palaearctic; wpal - Western Palaearctic; spal - Southern Palaearctic; eur - European; cosm - Cosmopolitan; wmed - Western Mediterranean; crim-adv - alien in Crimea.
leafPhenology_golubev	Leaf phenology (source: Golubev (1996)). Unit type: categorical (evergreen, summer-green, summer-winter-green, ephemeral, ephemeral-winter, ephemeral-spring). Explanation: evergreen - true evergreen plants; summer-green - deciduous plants which lose all of their leaves for part of the year; summer-winter-green - deciduous plants which can keep their leaves during the winter; ephemeral - annual ephemeral and late summer-autumn growing perennial ephemeral (ephemeroids); ephemeral-winter - winter-growing perennial ephemeral (ephemeroids); ephemeral-spring - spring-growing perennial ephemeral (ephemeroids).
rosette_golubev	Rosette plants (source: Golubev (1996)). Unit type: categorical (rosette, rosetteless, semi-rosette).
biomorphRoot_golubev	Root system (source: Golubev (1996)). Unit type: categorical (fibrous-short, fibrous-medium, fibrous-long, taproot-short, taproot-medium, taproot-long). Explanation: two types of root system - fibrous and taproot, which may be short (in the upper soil layer), medium or long (deep in the soil).
tree_golubev	Golubev life form: belonging to trees (source: Golubev (1996)). Unit type: binary (1 and 0).
shrub_golubev	Golubev life form: belonging to shrubs (source: Golubev (1996)). Unit type: binary (1 and 0).
lowShrub_golubev	Golubev life form: belonging to low shrubs (source: Golubev (1996)). Unit type: binary (1 and 0).
subshrub_golubev	Golubev life form: belonging to subshrubs (source: Golubev (1996)). Unit type: binary (1 and 0).
lowSubshrub_golubev	Golubev life form: belonging to low subshrubs (source: Golubev (1996)). Unit type: binary (1 and 0).
polycarpicHerb_golubev	Golubev life form: belonging to polycarpic herbs (source: Golubev (1996)). Unit type: binary (1 and 0).
perennialMonocarpicHerb_golubev	Golubev life form: belonging to perennial monocarpic herbs (source: Golubev (1996)). Unit type: binary (1 and 0).
springAnnual_golubev	Golubev life form: belonging to spring annuals (source: Golubev (1996)). Unit type: binary (1 and 0).
autumnAnnual_golubev	Golubev life form: belonging to autumn annuals (source: Golubev (1996)). Unit type: binary (1 and 0).
epihydrophyte_golubev	Golubev life form: belonging to epihydrophyte plants (aquatic plants with floating leaves on the surface) (source: Golubev (1996)). Unit type: binary (1 and 0).
idiohydrophyte_golubev	Golubev life form: belonging to idiohydrophyte plants (submerged aquatic plants) (source: Golubev (1996)). Unit type: binary (1 and 0).
liana_golubev	Golubev life form: belonging to lianas (source: Golubev (1996)). Unit type: binary (1 and 0).
sparseCushion_golubev	Golubev life form: belonging to sparse cushion-shaped plants (source: Golubev (1996)). Unit type: binary (1 and 0).
spheric_golubev	Golubev life form: belonging to spherical-shaped plants (source: Golubev (1996)). Unit type: binary (1 and 0).
creeping_golubev	Golubev life form: belonging to creeping plants (source: Golubev (1996)). Unit type: binary (1 and 0).
succulent_golubev	Golubev life form: belonging to succulents and fleshy plants (source: Golubev (1996)). Unit type: binary (1 and 0).
parasite_golubev	Golubev life form: belonging to parasitic plants (source: Golubev (1996)). Unit type: binary (1 and 0).
semiparasite_golubev	Golubev life form: belonging to semi-parasitic plants (source: Golubev (1996)). Unit type: binary (1 and 0).
saprophyte_golubev	Golubev life form: belonging to saprophytic plants (source: Golubev (1996)). Unit type: binary (1 and 0).
carnivorous_golubev	Golubev life form: belonging to carnivorous plants (source: Golubev (1996)). Unit type: binary (1 and 0).
rhizomatous_golubev	Golubev life form: belonging to rhizomatous plants (source: Golubev (1996)). Unit type: binary (1 and 0).
aboveBulbs_golubev	Golubev life form: belonging to plants with aboveground brood nodules and bulbs (source: Golubev (1996)). Unit type: binary (1 and 0).
undergroundBulbs_golubev	Golubev life form: belonging to plants with underground brood bulbs, corms and nodules (source: Golubev (1996)). Unit type: binary (1 and 0).
repStemTuber_golubev	Biomorphological adaptations for vegetative renewal and reproduction: belonging to plants with stem tubers (source: Golubev (1996)). Unit type: binary (1 and 0).
repRootTuber_golubev	Biomorphological adaptations for vegetative renewal and reproduction: belonging to plants with root tubers (source: Golubev (1996)). Unit type: binary (1 and 0).
repBulb_golubev	Biomorphological adaptations for vegetative renewal and reproduction: belonging to plants with bulbs (source: Golubev (1996)). Unit type: binary (1 and 0).
repTuft_golubev	Biomorphological adaptations for vegetative renewal and reproduction: belonging to plants with a dense tuft (source: Golubev (1996)). Unit type: binary (1 and 0).
repMediumRhizome_golubev	Biomorphological adaptations for vegetative renewal and reproduction: belonging to plants with a medium-size rhizome (source: Golubev (1996)). Unit type: binary (1 and 0).
repLongRhizome_golubev	Biomorphological adaptations for vegetative renewal and reproduction: belonging to plants with a long-size rhizome (source: Golubev (1996)). Unit type: binary (1 and 0).
repAbovegroundStolon_golubev	Biomorphological adaptations for vegetative regeneration and reproduction: belonging to plants with aboveground stolons (source: Golubev (1996)). Unit type: binary (1 and 0).
repUndergroundStolon_golubev	Biomorphological adaptations for vegetative renewal and reproduction: belonging to plants with underground stolons (source: Golubev (1996)). Unit type: binary (1 and 0).
repCreeping_golubev	Biomorphological adaptations for vegetative renewal and reproduction: belonging to creeping plants (source: Golubev (1996)). Unit type: binary (1 and 0).
rdbu2021	Listed in the Red Data Book of Ukraine (edition 2021). Unit type: binary (1 and 0). Only corresponds to a column taxonUkr (nomenclature based on Mosyakin and Fedoronchuk (1999)).
rdbuStatus	Conservation status according to the Red Data Book of Ukraine (edition 2021). Unit type: categorical (DD, EN EW, EX, NA, RR, UV, VU).
alienStatus	Residence time status of alien species according to Protopopova (1991) and Protopopova and Shevera (2015), with modification for neophyte (doubtfully). Unit type: categorical (archaeophyte, archaeophyte (doubtfully), NA, neophyte, neophyte (doubtfully).
regionOrigin	Region of origin of the alien species according to Protopopova (1991) and Protopopova and Shevera (2015). Unit type: categorical (Africa, Asia, Asia/Africa, Australia, Caucasus, Caucasus/Asia, Caucasus/Europe, Euro-Mediterranean, Europe, Europe(?), Europe/Asia, hybrid origin, Irano-Turanian, Mediterranean, Mediterranean/Asia, Mediterranean/Caucasus, Mediterranean-Irano-Turanian, NA, North America, North America/Asia, Pontic, South America, South Europe, Tropical, Tropical(America), not defined).
alienType	Naturalisation degree of the alien species according to Protopopova and Shevera (2015) and following Kornaś (1990). Unit type: categorical (agriophyte, colonophyte, ephemerophyte, epoecophyte, ergasiophigophyte, hemiepoecophyte).
cultivated_checklist1999	Cultivated plants according to Mosyakin and Fedoronchuk (1999). Unit type: categorical (cultivated, NA).
escaped_checklist1999	Escaped from cultivation plants according to Mosyakin and Fedoronchuk (1999). Unit type: categorical (escaped, NA).

### Data set 3.

#### Data set name

Taxonomical crosswalks between the UkrTrait species list and other nomenclature sources

#### Description

Dataset is available in Suppl. material 3.

**Data set 3.** 

Column label	Column description
ID_UkrTrait	ID of the species in the UkrTrait database.
taxon_UkrTrait	Taxon name in the UkrTrait database.
authorship_UkrTrait	Taxon authorship in the UkrTrait database.
taxonLevel_UkrTrait	Taxonomic level of the taxon in the UkrTrait database: species, subspecies, variety, hybrid.
taxonGenus_UkrTrait	Taxon genus in the UkrTrait database.
taxonFamily_UkrTrait	Taxon family in the UkrTrait database according to Euro+Med PlantBase.
scientificName_checklist1999	Taxon name in the Ukrainian Checklist (Mosyakin and Fedoronchuk 1999).
authorship_checklist1999	Taxon author in the Ukrainian Checklist (Mosyakin and Fedoronchuk 1999).
scientificName_euroPlusMed	Name of the corresponding accepted taxon in the Euro+Med PlantBase.
authorship_euroPlusMed	Authorship of the corresponding accepted taxon in the Euro+Med PlantBase.
scientificName_gbif	Taxon name according to GBIF Backbone Taxonomy.
rank_gbif	Taxonomic rank of the taxon in GBIF Backbone Taxonomy: SPECIES, SUBSPECIES, VARIETY.
key_gbif	Taxon ID (key) in the GBIF Backbone Taxonomy.
matchType_gbif	Taxonomic match type in the GBIF Species Lookup Tool: EXACT (for species with exact match) and EDITED (for species matched manually).
status_gbif	Taxon status in the GBIF Backbone Taxonomy: ACCEPTED, DOUBTFUL, PROPARTE_SYNONYM, SYNONYM.
acceptedUsageKey_gbif	Taxon ID (key) of the corresponding accepted taxon in the GBIF Backbone Taxonomy.
name_wcvp	Taxon name according to the World Checklist of Vascular Plants (WCVP).
authors_wcvp	Taxon authorship according to the WCVP.
status_wcvp	Taxonomic status according to the WCVP: Accepted, Artificial Hybrid, Illegitimate, Invalid, Local Biotype, Orthographic, Synonym, Unplaced.
id_wcvp	Taxon ID in the World Checklist of Vascular Plants (WCVP).
ipni_id_wcvp	Taxon ID of the taxon in the International Plant Name Index (IPNI) obtained through the WCVP.
accepted_id_wcvp	Taxon ID of the corresponding accepted taxon in the WCVP.
scientificName_wfo	Name of the corresponding accepted taxon in the World Flora Online (WFO).
scientificNameAuthorship_wfo	Authorship of the corresponding accepted taxon in the WFO.
taxonID_wfo	Taxon ID in the WFO.
taxonomicStatus_wfo	Taxonomic status according to the WFO: Accepted, Unchecked.

## Additional information

Integration and harmonisation of trait data from heterogeneous sources is an important task, given the rapid emergence of new databases around the world (Lenters et al. 2021). To facilitate the further use of our database, we provided terminological and nomenclature crosswalks.

### Harmonisation of nomenclature data and linking taxa names to the international taxonomical sources

We used the Ukrainian Checklist (Mosyakin and Fedoronchuk 1999) as a primary taxonomical source to preserve the original taxa names and their corresponding trait values, which is especially meaningful for the traits collected from the existing literature sources. In case of nomenclatural changes, lumping and splitting, the original interpretation of traits for certain species of Ukrainian flora will be preserved.

Since the Ukrainian Checklist was published more than 20 years ago, we added 110 species that were listed for the territory of Ukraine since the 2000s. In particular, these were alien species (e.g. *Elodeanuttallii*, *Opuntiaficus-indica* and *Persicariapensylvanica*), newly-described taxa (e.g. *Chenopodiumucrainicum* and ×*Dactylocamptisuechtritziana*) and other taxa newly listed for the territory of Ukraine (e.g. *Epipactisalbensis*, *E.tallosii*, *Torilispseudonodosa* and *Trichophorumalpinum*). After checking the taxa names and their authorships using the World Checklist of Vascular Plants (World Checklist of Vascular Plants (World Checklist of Vascular Plants (WCVP), Euro+Med PlantBase and International Plant Name Index (IPNI), we corrected misprints in 251 names to avoid misinterpretations and technical difficulties (Suppl. material 3).

Additionally, we provided the crosswalks between the Ukrainian checklist and international sources: GBIF Backbone Taxonomy, World Checklist of Vascular Plants (World Checklist of Vascular Plants (World Checklist of Vascular Plants (WCVP), World Flora Online (WFO) and Euro+Med PlantBase (Suppl. material 3). Using complete taxa names with authorship, we significantly improved the automatic matches, but names with fuzzy matches required additional expert review and corrections. However, the provided nomenclature crosswalks should be used with caution, since online databases are constantly updated. Therefore, we recommend conducting an additional match of the original taxa names (in accordance with the tools specified in the methodology of this paper) to obtain up-to-date nomenclature information.

### Linking trait categories with ontologies and other trait databases

We linked the trait terminology used in the UkrTrait to the Thesaurus of Plant Characteristics (TOP), the Plant Trait Ontology (TO) and the TRY Plant Trait Database (Table 1, Suppl. material 4). Almost all measured numerical traits had corresponding categories in the TRY Database and terms in the ontologies, except for 'Generative plant height measured in the field' and 'Vegetative plant height measured in the field', which were absent in the Plant Trait Ontology (TO). On the contrary, for the categorical traits, we did not find correspondences for the most of traits in the ontologies, while TRY Database had these trait categories.

### Further use and development of UkrTrait database

The UkrTrait database (version 1.0) represents a starting point for organising and measuring various plant traits in Ukraine. While our current version covers a significant range of traits, certain useful traits, for example, plant pollination syndromes, dispersal systems and rooting depth, documented in national literature sources have not yet been incorporated. In addition, it is important to continue measuring plant traits for species currently absent from or insufficiently covered by other trait databases. This involves a specific focus on endemic and rare species, as well as on species common in Ukraine, but rare in other parts of Europe, particularly steppic plants. The current version of the database is uploaded to the Zenodo repository, where we also plan to release updated versions. Looking ahead, we want the UkrTrait database not only to keep growing, but also to connect globally. This would include integrating our database into the global TRY Plant Trait Database (Kattge et al. 2020) and into the pan-European project FloraVeg.EU, an online database of European vegetation, habitats and flora (Chytrý et al. 2024).

## Supplementary Material

2C840BB9-1903-5A4B-908A-724DA32B9406 10.3897/BDJ.12.e118128.suppl1 Supplementary material 1 Dataset of plant traits from literature sources Data type literature trait data (csv format). File: oo_976942.csv https://binary.pensoft.net/file/976942 Denys Vynokurov, Dariia Borovyk, Olha Chusova, Anastasia Davydova, Denys Davydov, Iwona Dembicz, Svitlana Iemelianova, Ganna Kolomiiets, Anna Kuzemko, Ivan Moysiyenko, Viktor Shapoval, Oleksandr Shynder, Nadiia Skobel.

A5B93F0B-101C-5C73-9384-D2525DFF5641 10.3897/BDJ.12.e118128.suppl2 Supplementary material 2 Dataset of measured plant traits Data type laboratory and field trait measurements (csv format). File: oo_960822.csv https://binary.pensoft.net/file/960822 Denys Vynokurov, Dariia Borovyk, Olha Chusova, Anastasia Davydova, Ivan Moysiyenko, Viktor Shapoval, Nadiia Skobel.

D89EA0FB-66FD-586F-BE6A-FD917498BF0A 10.3897/BDJ.12.e118128.suppl3 Supplementary material 3 Nomenclature crosswalks between the UkrTrait species list and selected checklists Data type species checklist (csv format, UTF-8 encoding). File: oo_960823.csv https://binary.pensoft.net/file/960823 Dariia Borovyk, Jiří Danihelka, Denys Vynokurov

1EFCD7CD-D38C-5E52-8C4B-45B16E7358B9 10.3897/BDJ.12.e118128.suppl4 Supplementary material 4 Links for trait terms between UkrTrait, TRY database and trait ontologies (TOP, TO) Data type table with terminological crosswalks (csv format, UTF-8 encoding). File: oo_976946.csv https://binary.pensoft.net/file/976946 Dariia Borovyk

## Figures and Tables

**Table 1.** Main numerical and categorical traits from the Ukrainian Trait Database (Suppl. material 1 and Suppl. material 2) and corresponding names and identifiers from the TRY Plant Trait Database and trait ontologies (full version of this table with additional traits is given in the Suppl. material 4). Columns: "UkrTrait" - traits from the Ukrainian Trait Database; "unit" - measurement unit; "nameTRY" and "idTRY" - connection to the TRY Database (trait name and id, respectively), "formalNameTOP" and "idTOP" - to the Thesaurus of Plant Characteristics (TOP), "nameTO" and "idTO" - to the Plant Trait Ontology (TO). Empty fields indicate that the respective trait name had no source in the TRY, TO or TOP.

**UkrTrait**	**unit**	**nameTRY**	**idTRY**	**formalNameTOP**	**idTOP**	**nameTO**	**idTO**
Generative plant height measured in the field	cm	Plant height generative	3107	Whole plant height generative	TOP70		
Vegetative plant height measured in the field	cm	Plant height vegetative	3106	Whole plant height vegetative	TOP69		
Leaf area - an average one-sided projected surface area of the fresh leaf	mm^2^	Leaf area (in case of compound leaves: leaf, petiole included)	3110	Leaf area	TOP25	Leaf area trait	TO:0000540
Average dry leaf mass	mg	Leaf dry mass (single leaf)	55	Leaf dry mass	TOP40	Leaf dry weight	TO:0001014
Average specific leaf area (SLA) - average one-sided area of the fresh leaf divided by its oven-dry mass	mm^2^/mg	Leaf area per leaf dry mass (specific leaf area, SLA or 1/LMA): petiole included	3116	Leaf area per leaf dry mass	TOP50	Specific leaf area	TO:0000562
Leaf nitrogen concentration - a ratio of the quantity of nitrogen in the leaf per respective unit of dry mass	mg/g	Leaf nitrogen (N) content per leaf dry mass	14	Leaf nitrogen content per leaf dry mass	TOP462	Leaf nitrogen content	TO:0000543
Leaf phosphorus concentration - a ratio of the quantity of phosphorus in the leaf per respective unit of dry mass	mg/g	Leaf phosphorus (P) content per leaf dry mass	15	Leaf phosphorus content per leaf dry mass	TOP463	Leaf phosphorus content	TO:0001025
Length of a seed	mm	Seed length	27	Seed length	TOP91	Seed length	TO:0000146
Width of a seed	mm	Seed width	239	Seed width	TOP95	Seed width	TO:0000149
Thickness of a seed	mm	Seed thickness	238	Seed thickness	TOP99	Seed thickness	TO:0000304
Seed shape - variance of three dimensions of a seed	unitless (0-1)	Seed shape	349	Seed shape	TOP114	Seed shape	TO:0000484
Seed mass - average dry mass of a seed	mg	Seed dry mass	26	Seed dry mass	TOP111	Seed weight	TO:0000181
Plant height - the average height of a whole plant	cm	Plant height	18	Whole plant height	TOP68	Plant height	TO:0000207
Plant phenology - flowering in each of the 12 months (Jan-Dec)	binary (1/0) for each month	Plant reproductive phenology timing (flowering time)	335				
Plant life span - belonging to annuals, biennials or short-lived plants, perennials	binary (1/0) for each category	Plant lifespan (longevity)	59			Life cycle habit	TO:0002725
Raunkiaer life form	categorical and binary (1/0) for each category	Plant life form (Raunkiaer life form)	343	Whole plant life form	TOP210		
Plant growth form	categorical and binary (1/0) for each category	Plant growth form	42	Whole plant growth form	TOP136		
Status of alien species	categorical	Species occurrence range characteristics	1140				
